# Probit Models to Investigate Prevalence of Total Diagnosed and Undiagnosed Diabetes among Aged 45 Years or Older Adults in China

**DOI:** 10.1371/journal.pone.0164481

**Published:** 2016-10-10

**Authors:** Minghui Yin, Balekouzou Augustin, Chang Shu, Tingting Qin, Ping Yin

**Affiliations:** Department of Epidemiology and Biostatistics, School of Public Health, Tongji Medical College, Huazhong University of Science and Technology, Wuhan, Hubei, China; Medical University Innsbruck, AUSTRIA

## Abstract

The aims of this study are to identify the most important predictors of total diagnosed and undiagnosed diabetes and estimate the mean change in the predicted probability among aged 45+ adults in China. We used baseline data collected from 2011 wave of the China Health and Retirement Longitudinal Study (CHARLS) (n = 9,513). First, we estimated the prevalence of diagnosed, measured, total diagnosed, and undiagnosed diabetes. Second, we used probit models to determine whether individual attributes, socioeconomic characteristics and behavioral health factors, including smoking, alcohol consumption, obesity, central obesity, are associated with total diagnosed and undiagnosed diabetes. We also consider other factors, including contact with medical system, hypertension and urban/rural settings. Third, we estimated average marginal effects of variables in probit models. Among Chinese people aged 45+, the prevalence of diagnosed, measured, total diagnosed and undiagnosed diabetes were 5.8% (95%CI, 5.3%-6.3%), 14.7% (95%CI, 14.0%-15.4%), 17.0% (95%CI, 16.3%-17.7%), 11.3% (95%CI, 10.6%-12.0%), respectively. The probability of total diagnosed diabetes is 3.3% (95% CI, 1.2%-5.3%) and 10.2% (95% CI, 7.0%-13.5%) higher for overweight and obesity than normal BMI, 5.0% (95% CI, 3.0%-7.1%) higher for central obesity than normal waist circumference, 5.4% (95% CI, 3.7%-7.0%) higher for hypertensive than normotensive and 1.8% (95% CI, 0.8%- 2.7%) higher in urban areas than in rural areas, respectively. The probability of undiagnosed diabetes is 2.7% (95% CI, 1.2%-4.2%) and 7.2% (95% CI, 4.7%-9.6%) higher for overweight and obesity than normal BMI, 2.6% (95% CI, 0.9%-4.4%) higher for central obesity than normal waist circumference and 2.6% (95% CI, 1.2%-4.0%) higher for hypertensive than normotensive, respectively, and -1.5% (95% CI, -2.5% to -0.5%) lower for individuals who were in contact with the medical system. Greater focus on prevention of diabetes is necessary for obesity, central obesity, hypertensive and in urban areas for middle-aged and older in China.

## Introduction

Cardiovascular disease has become the leading cause of death in China, a development that has followed rapid economic growth, an increase in life expectancy, and changes in lifestyle.[1] Diabetes is one of the leading causes of mortality and morbidity worldwide, which increases the risk for cardiovascular and kidney diseases[2], and the prevalence of diabetes is high and increasing in China.[3–6]

In China, approximately 114 million, or 11.6% of adults, had diabetes in 2010.[6] However, less than one-third (30.1%) of these patients were aware of their disease condition[6], and this may affect the estimate of diabetes prevalence. The management of diabetes in China has been ineffective for many years.[7] Although these studies have documented a marked increase in the prevalence of diabetes in China and many researchers have studied the risk factors related to diabetes, there has been little investigation to study risk factors leading to the effects of higher probability of diabetes.

In many survey studies with a component or a focus on health, prevalence of diabetes is according to "self-reports": respondents are asked to report whether they have diabetes at present and/or have ever been diagnosed with diabetes. However, the actual prevalence, based on self-reports, might be seriously underestimated. This is because the signs and symptoms of type 1 diabetes are usually obvious and develop very quickly, often over a few weeks, but type 2 diabetes tends to develop more slowly, usually over a period of months or even years, and prevalence of undetected or undiagnosed diabetes are generally high.[8]

An effective way to assess the extent to which diabetes is undiagnosed is to collect both objective measures and self-reported for the same respondents. This is, however, rarely done in survey studies. China Health and Retirement Longitudinal Study (CHARLS) collected both of the information.

Our first aim is to identify the most important factors leading to higher probability of total diagnosed and undiagnosed diabetes using baseline data from a national survey among aged 45+ adults in China. Our second aim is to estimate the mean change in the predicted probability.

## Methods

### Data

We used data collected from the CHARLS of Chinese people aged 45 years or older.[9, 10] CHARLS, harmonized with the U.S. Health and Retirement Study (HRS) family of surveys, is publicly available and de-identified.[10] The data were collected in a survey in which four-stage, stratified, cluster sampling was used to select eligible individuals. A total of 17,708 individual participants (10,069 aged 45 years or older and 7,639 spouses of eligible individuals) completed a computer-assisted personal interview (CAPI) in their home.[9–11] A structured questionnaire with several main sections was used to collect data from each respondent. Some demographical variables were gathered by a face-to-face interview. As explained in detail by Zhao et al.[9]

For our analysis, we used the first wave (2011) of CHARLS which was conducted between June 2011 and March 2012 among aged 45 years or older adults in China. Information on various aspects of the respondents' lives were collected, including individual characteristics, behavioral health, health status, the economic dimension, the social domain, blood pressure measurements and urban/rural settings. Also, measurement of blood glucose and/or HbA1c was collected. Of 17,708 respondents, 6,173 were not measured glucose and glycosylated hemoglobin, 101 without self-reported diabetes answers, 40 without measured blood pressure, 22 missing gender and education, 1,620 without BMI and waist circumference measurements, 239 without smoking and drinking answers. The final sample size was 9,513 for our study. The coded data is available in S1 Table.

### Measures of diabetes prevalence

#### Diagnosed

CHARLS collected information on individual self-reports of specific conditions with the general question: " Have you been diagnosed with diabetes or high blood sugar by a doctor?". We classified respondents as having "diagnosed diabetes" if they answered "yes" to the questions.

#### Measured

The key advantage in using data collected from CHARLS is that blood samples were measured in the survey. Nearly two-thirds blood samples of individuals were collected by medically trained staff from the China Center for Disease Control and Prevention. Participants were asked to fast overnight. After collection, plasma for glucose assay was separated from blood samples and stored at -20°C,[10] and whole blood for HbA1C assay was stored immediately and during shipment at 4°C.[10] All the blood samples were transported within 2 weeks to the China Center for Disease Control and Prevention, where samples were placed at -80°C in a deep freezer before assay.[10] Blood assays were performed at the Youanmen Center for Clinical Laboratory of Capital Medical University during February 2013 and June 2013.[10] The laboratory used quality control samples daily during the testing of the CHARLS study samples, and all test results were within the target range (within two SDs of mean quality control concentrations). Glucose was measured using an enzymatic colorimetric test, and HbA1c was analyzed using boronate affinity chromatography.[10]

We used a binary variable for measured diabetes according to measurement of blood glucose and/or HbA1c. We divided respondents as diabetics if their fasting plasma glucose ≥ 126 mg/dL and/or HbA1c ≥ 6.5%. The cut-off points for diagnosis of diabetes were based on current recommendations from the American Diabetes Association.[12]

#### Total diagnosed

The total diagnosis coded respondents as diabetics if either they self-reported to be diabetic and/or the measured value above the diagnostic threshold, which is fasting plasma glucose ≥ 126 mg/dL and/or HbA1c ≥ 6.5%.

#### Undiagnosed

We divided respondents as having "undiagnosed diabetes" if they did not report having been told by a doctor that they have diabetes but were diabetics according to the more comprehensive total diagnosis. The prevalence of undiagnosed diabetes is the fraction of total prevalence that is not diagnosed.

#### Control variables

Our study included age, gender and marital status as individual attributes. We used two variables of socioeconomic status: level of education and household income. We classified level of education as illiterate, primary education, secondary education and at least college level based on Chinese education system. As for the income, we employed information from a single comprehensive question about income according to all household members. We corrected household income via dividing it by the square root of the number of persons in the household.[13–15] Then, we assigned individuals into corresponding income tertiles: first tertile (low income), second tertile (middle income) and third tertile (high income).[16]

CHARLS collected information on several health-related behaviors. We identified three categories for smoking: current smoker, past smoker and never smoked according to the respondents' tobacco use answer; and respondents were grouped into three categories of drinking—never, less than once a month, more than once a month according to according to their alcohol consumption answer.

The survey also collected information on body mass index (BMI), which is the ratio of weight in kilograms to height in meters squared. We use BMI to identify whether respondents are underweight/normal (BMI < 23), overweight (BMI ≥ 23 and < 27.5) and obese (BMI ≥ 27.5) based on the suggested categories for Asian populations.[17]

We defined the respondents as central obesity according to the male's waist circumference ≥ 90cm or the female's waist circumference ≥ 80cm based on the International Diabetes Federation suggested categories for Chinese populations.[18]

We also included a variable meant to capture the extent of "contact with the medical system". We created a dichotomous variable equal to one if the respondent had visited a public hospital, private hospital, public health center, clinic, or health worker's or doctor's practice, or been visited by a health worker or doctor for outpatient care or having received inpatient care at least once.

We coded respondents as hypertensive if either they self-reported to be hypertensive and/or had blood pressure value above the diagnostic threshold, which is SBP ≥ 140 mmHg and/or DBP ≥ 90 mmHg.

We also considered each respondent's urban/rural settings according to he or she was recorded as a long-term urban resident or long-term rural resident.

Although other risk factors might be important, we used some key variables based on other studies in our study.

### Statistical methods

We used unweighted probit models of determinants of total diagnosed and undiagnosed diabetes prevalence. We used R version 3.2.3 (R Core Team 2015, Vienna, Austria) to conduct the analyses[19] and set a prior level of significance at 0.05. We transformed the parameter estimates of probit models to estimates of average marginal effects AMEs.[20–23] AMEs are an effective means by which the effects of variables in nonlinear models can be made more intuitively meaningful.[24] Briefly, the AMEs of a categorical variable is the mean change in the predicted probability that the outcome is equal to one as the categorical variable changes from 0 to 1, holding all other covariates at their observed values.[25]

## Results

### Baseline characteristics

Table 1 listed the prevalence of diagnosed, measured, total diagnosed and undiagnosed diabetes and 95% confidence intervals. The prevalence of diagnosed, measured, total diagnosed and undiagnosed diabetes were 5.8% (95%CI, 5.3%-6.3%), 14.7% (95%CI, 14.0%-15.4%), 17.0% (95%CI, 16.3%-17.7%), 11.3% (95%CI, 10.6%-12.0%), respectively. The total prevalence of diabetes is high in China. Table 1 also indicated levels in key diabetes risk factors.

**Table 1:** Diabetes prevalence rates and diabetes risk factors in China (2011–2012): 45+ years old.

	China (%)
Diabetes prevalence	
Diagnosed	5.8 (5.3, 6.3)
Measured	14.7 (14.0, 15.4)
Total diagnosed	17.0 (16.3, 17.7)
Undiagnosed	11.3 (10.6, 12.0)
Individual characteristics	
Age, mean	59.2
Male	45.7 (44.7, 46.7)
Married	87.7 (87.1, 88.3)
Behavioural health	
Smoking	
Non-smoker	61.4 (60.4, 62.4)
Past smoker	8.7 (8.2, 9.2)
Current smoker	29.9 (28.9, 30.9)
Alcohol consumption^a^	
Abstainer	67.6 (66.7, 68.5)
Current drinker (>0 but ≤1)	7.8 (7.3, 8.3)
Current drinker (>1)	24.5 (23.7, 25.3)
BMI category	
Under/normal weight	48.3 (47.3, 49.3)
Overweight	37.7 (36.7, 38.7)
Obesity	14.0 (13.4, 14.6)
Waist Circumference	
Normal waist circumference	48.7 (47.7, 49.7)
Central obesity	51.3 (50.3, 52.3)
Socioeconomic gradient	
Education	
Illiterate	28.9 (28.0, 29.8)
Primary	41.5 (40.5, 42.5)
Secondary	28.3 (27.4, 29.2)
College and above	1.3 (1.1, 1.5)
Adjusted household income^b^	
First tercile	33.4 (32.4, 34.4)
Second tercile	35.3 (34.3, 36.3)
Third tercile	31.4 (30.4, 32.4)
Medical system	
Not contact with medical system	72.0 (71.1, 72.9)
Contact with medical system	28.0 (27.1, 28.9)
Blood pressure^c^	
Normotension	59.2 (58.2, 60.2)
Hypertension	40.8 (39.9, 41.7)
Urban/rural settings	
Rural area	65.0 (64.1, 65.9)
Urban area	35.0 (34.0, 36.0)
N	9,513

*BMI* body mass index

^a^ Drinking episodes per month.

^b^ First tertile (low income), second tertile (middle income) and third tertile (high income).

^c^ Hypertensive, if either they self-reported to be hypertensive and/or had a blood pressure value above the diagnostic threshold, which is SBP ≥ 140 mmHg and/or DBP ≥ 90 mmHg.

### Predictors of diabetes prevalence

**Fig 1:**
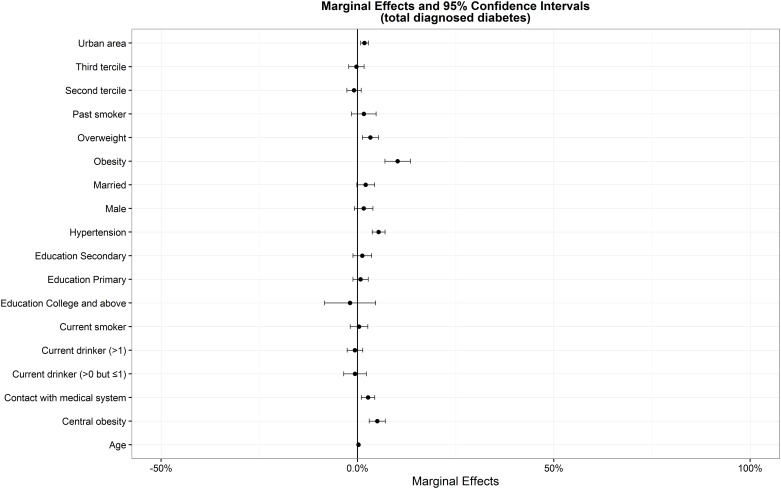
Average marginal effects and 95% confidence intervals from probit models for the prevalence of total diagnosed diabetes.

**Fig 2:**
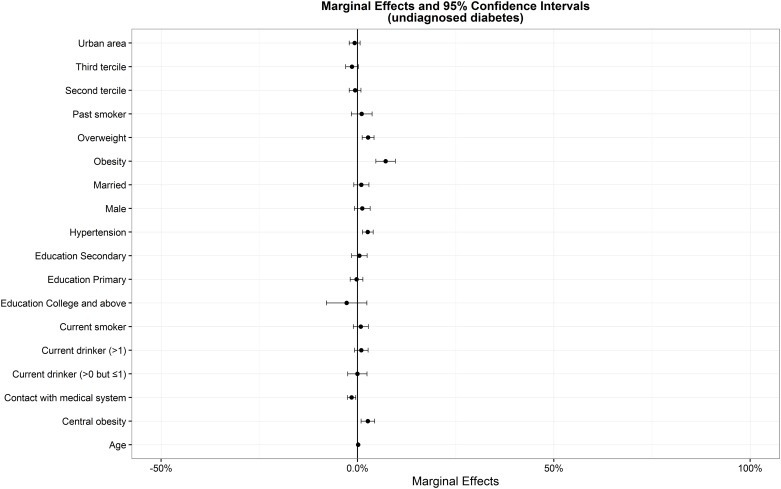
Average marginal effects and 95% confidence intervals from probit models for the prevalence of undiagnosed diabetes.

Table 2 listed estimates of probit models for total diagnosed and undiagnosed diabetes prevalence. The model has two functions. The first is to identify the key factors leading to higher probability of total diagnosed and undiagnosed diabetes, and the second is to estimate AMEs of variables in probit models (Table 2, Figs 1 and 2).

**Table 2:** Average marginal effects (95% confidence intervals) in probit models for diabetes prevalence (total diagnosed and undiagnosed) in China: 45+ years old.

	Total diagnosed		Undiagnosed	
	A.M.E. (95% CI) (%)	p	A.M.E. (95% CI) (%)	p
Individual characteristics				
Age	0.3 (0.2, 0.3)	0.000***	0.2 (0.1, 0.2)	0.000***
Male	1.6 (-0.8, 3.9)	0.192	1.2 (-0.8, 3.2)	0.236
Married	2.1 (-0.2, 4.4)	0.070	1.0 (-0.9, 2.9)	0.317
Behavioral health				
Smoking				
Non-smoker	Ref.		Ref.	
Past smoker	1.6 (-1.5, 4.7)	0.313	1.1 (-1.6, 3.7)	0.426
Current smoker	0.4 (-1.9, 2.6)	0.744	0.9 (-1.1, 2.8)	0.382
Drinking				
Abstainer	Ref.		Ref.	
Current drinker (>0 but ≤1)	-0.6 (-3.5, 2.2)	0.670	0.0 (-2.5, 2.4)	0.982
Current drinker (>1)	-0.7 (-2.7, 1.3)	0.518	1.0 (-0.8, 2.7)	0.272
BMI category				
Under/normal weight	Ref.		Ref.	
Overweight	3.3 (1.2, 5.3)	0.002**	2.7 (1.2, 4.2)	0.000***
Obesity	10.2 (7.0, 13.5)	0.000***	7.2 (4.7, 9.6)	0.000**
Waist Circumference				
Normal waist circumference	Ref.		Ref.	
Central obesity	5.0 (3.0, 7.1)	0.000***	2.6 (0.9, 4.4)	0.003**
Health status				
Medical system				
Not contact with medical system	Ref.		Ref.	
Contact with medical system	2.7 (1.0, 4.4)	0.002**	-1.5 (-2.5, -0.5)	0.005**
Blood pressure				
Normotension	Ref.		Ref.	
Hypertension	5.4 (3.7, 7.0)	0.000***	2.6 (1.2, 4.0)	0.000***
Socioeconomic gradient				
Education				
Illiterate	Ref.		Ref.	
Primary	0.8 (-1.2, 2.7)	0.434	-0.2 (-1.9, 1.4)	0.773
Secondary	1.2 (-1.2, 3.6)	0.315	0.5 (-1.5, 2.5)	0.638
College and above	-1.9 (-8.4, 4.6)	0.568	-2.8 (-7.9, 2.4)	0.294
Adjusted household income				
First tercile	Ref.		Ref.	
Second tercile	-0.9 (-2.7, 0.9)	0.336	-0.6 (-2.1, 0.9)	0.423
Third tercile	-0.3 (-2.2, 1.7)	0.784	-1.4 (-3.0, 0.2)	0.087
Urban/rural settings				
Rural area	Ref.		Ref.	
Urban area	1.8 (0.8, 2.7)	0.000***	-0.7 (-2.1, 0.7)	0.309

*AME* average marginal effect, *CI* confidence interval, *REF* reference category the AME of a categorical variable is the mean change in the predicted probability that the outcome is equal to one as the categorical variable changes from 0 to 1, holding all other covariates at their observed values. To illustrate, the probability of being measured is 4.6 percentage points higher for Central obesity than Normal waist circumference (95% CI, 2.8–6.4), holding all other covariates at their observed values.

* p < 0.05,

** p < 0.01,

*** p < 0.001

In China, for 45+ adults, age, overweight or obesity, central obesity, "contact with the medical system", hypertension and urban area were positively associated with the probability of total diagnosed diabetes. The probability of being diabetic based on the more comprehensive total diagnosis was higher for individuals who were older, overweight or obese, central obesity, contact with the medical system, hypertensive and living in urban area.

Age, overweight or obesity, central obesity and hypertension were positively associated with the probability of undiagnosed diabetes. "Contact with the medical system" was negatively associated with the probability of undiagnosed diabetes. The probability of undiagnosed diabetes was higher for who were older, overweight or obese, central obesity and hypertensive, and lower for individuals who were contact with the medical system.

A number of differences in the sign or significance of diabetes risk factors also emerged. Age had significant influence on diabetes. Notably, the probability of total diagnosed diabetes is 3.3% (95% CI, 1.2%-5.3%) and 10.2% (95% CI, 7.0%-13.5%) higher for overweight and obesity than normal BMI, 5.0% (95% CI, 3.0%-7.1%) higher for central obesity than normal waist circumference, 5.4% (95% CI, 3.7%-7.0%) higher for hypertensive than normotensive and 1.8% (95% CI, 0.8%- 2.7%) higher for in urban areas than in rural areas, respectively. The probability of undiagnosed diabetes is 2.7% (95% CI, 1.2%-4.2%) and 7.2% (95% CI, 4.7%-9.6%) higher for overweight and obesity than normal BMI, 2.6% (95% CI, 0.9%-4.4%) higher for central obesity than normal waist circumference and 2.6% (95% CI, 1.2%-4.0%) higher for hypertensive than normotensive, respectively, and -1.5% (95% CI, -2.5% to -0.5%) lower for individuals who were contact with the medical system.

**Fig 3:**
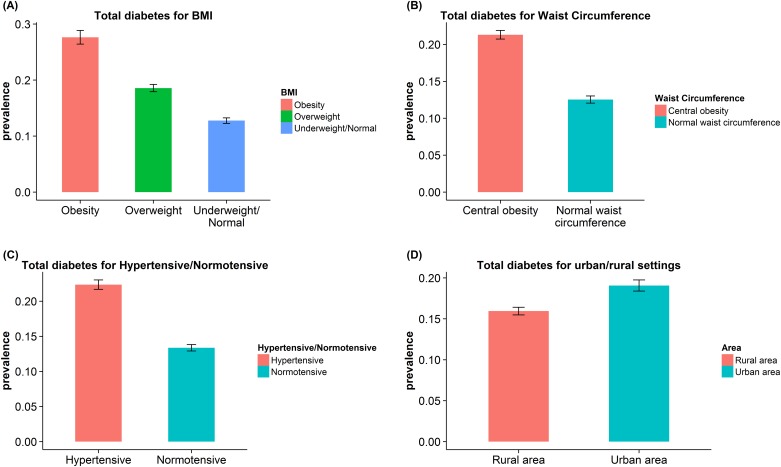
Comparisons of the prevalence of total diabetes by BMI, waist circumference, blood pressure and urban/rural settings.

To investigate further on the four key factors of the results discovered by the probit models: BMI, waist circumference, hypertension and urban/rural settings. We compared the prevalence of total diagnosed diabetes for each group (Fig 3). The prevalence of total diagnosed diabetes for underweight/normal, overweight and obesity were 12.8%, 18.6% and 27.7%, respectively. The prevalence of total diagnosed diabetes for normal waist circumference and central obesity were 12.5% and 21.3%, respectively. The prevalence of total diagnosed diabetes for normotensive and hypertensive were 13.4% and 22.4%, respectively. The prevalence of total diagnosed diabetes for rural area and urban area were 15.9% and 19%, respectively.

## Discussion

Probit models is an importance as a method to determine key factors on public health and used to estimate AMEs of variables. In this study, using CHARLS data on ageing, we took advantage of AMEs in probit models to investigate the prevalence of total diagnosed and undiagnosed diabetes among Chinese people aged 45 years or older. We identified the most important predictors of diabetes leading to higher probability of diabetes through probit models. It is worth noting that diabetes is a major risk factor for cardiovascular disease, and the prevalence of diabetes is high and is increasing in China. China has also strengthened monitoring and adopted diabetes prevention strategies.

Our results show that, based on the more comprehensive total diagnosis that combines diagnosed and measured diabetes, the prevalence of diabetes is high in China. Six results are outstanding to the predictors of diabetes prevalence for marginal effects in probit models.

First, age is positively associated with the probability of total diagnosed and undiagnosed diabetes, respectively. This finding is in line with many studies[26, 27], as they confirm increasing age was an important risk factor for diabetes.

Second, obesity and central obesity are positively associated with total diagnosed and undiagnosed diabetes, respectively. Being overweight or obese is the main modifiable risk factor for type 2 diabetes, and a large waist circumference is associated with increased likelihood of developing type 2 diabetes.[28] Both a high proportion of body fat and a predominance of central obesity are associated with insulin resistance.[29, 30] The precise mechanisms linking central obesity to insulin resistance remain, however, unclear.

Third, "contact with the medical system" is positively associated with the probability of total diabetes and negatively associated with the probability of undiagnosed diabetes. It may be that the more visits are, the more opportunity physicians detecting undiagnosed diabetes will be, or the causality may be reversed, and those who have already been diagnosed make more visits to monitor treatment effectiveness or refill prescriptions.

Fourth, hypertension is positively associated with total diagnosed and undiagnosed diabetes, respectively. A meta-analysis shows that a high blood pressure could increase the risk of developing type 2 diabetes by around 50%.[31] And there is evidence to suggest that lowering blood pressure significantly reduces diabetes related deaths, strokes, heart failure and microvascular complications.[32, 33] It may be that diabetes and hypertension share common pathways such as sympathetic nervous system, renin-angiotensin-aldosterone system, oxidative stress, adipokine, insulin resistance, and peroxisome proliferator-activated receptors. These pathways interact and influence each other and may even cause a vicious cycle. Hypertension and diabetes are both end results of the metabolic syndrome.[34]

Fifth, the probability of total diabetes is higher in urban areas than in rural ones. This finding is similar to zhou' study[35], which using geographic visualization at the provincial level indicated widespread variation in diabetes prevalence and detection across China. Urban people are more likely to suffer from diabetes than rural ones, the prevalence of diabetes is13.1% in urban areas, while 8.7% in rural areas.[35] Peng et al.[36] investigated the relationship between changing lifestyles and non-communicable disease, and they found urbanization has led to changes in patterns of human activity, diet, and social structures in China, with pro-found implications for non-communicable, including diabetes.[36] He and colleagues' study[37] in a geographically and socially isolated ethnic minority group in southwest China provided early evidence of the effect of urbanization on chronic disease.[37] Since then, researchers have suggested that Chinese urban environments promote lifestyles that place people at risk of diabetes, obesity and central obesity.

Finally, education did not play a significant role in predicting the prevalence of total diagnosed and undiagnosed diabetes. A possible explanation is that the higher level of education completed is not correlated with obtaining medical knowledge regarding the prevention and treatment of diabetes. This suggests that educational programs to increase the awareness of diabetes and associated chronic diseases should target adults across all education levels.[38]

Turning to boronate affinity chromatography we used, it is possible that the values of HbA1c obtained from the samples which were frozen upon arrival in Beijing are lower than true values. Amount of studies reported that frozen samples can be used, but it is not the standard clinical laboratory practice. A number of studies have shown that the results of HbA1c assays which use the affinity high performance liquid chromatography (HPLC) method are stable on whole blood specimens that have been stored at -70°C for more than 2 decades.[39] However, it has been shown that the results can be affected by the ambient conditions and the length of time whole blood samples are stored during transport from the field to the lab.[40] Storage at freezing temperature has also been related to assay value; the average frozen HbA1c was lower than fresh samples after a year, and the relative error ratio between HbA1c from frozen samples and from fresh samples was significantly higher at high levels of fresh HbA1c.[41]

Our findings should be considered in the context of several limitations.

First, the data analyses are the first wave for CHARLS, we investigated associations and were unable to confidently identify the causes of prevalence of diabetes.

Second, the measured individual attributes and risk factors are incomplete. CHARLS have not collected information on respondents' dietary patterns, which may offer additional explanatory power.

Third, only a random subsample of households was asked about the amount of time you spend on different types of physical activities. Because of this, we could not adequately analyze the effects of physical activity contribute to the outcomes.

Despite these limitations, it is noteworthy that this study is unique in using the survey data by probit models to identify the key factors leading to higher probability of total diagnosed and undiagnosed diabetes, which provided useful information on health outcome for Chinese people aged 45 years or older.

In conclusion, our results indicate that the probability of total diagnosed diabetes among Chinese people aged 45 years or older adults are significantly higher for obesity than normal BMI, higher for central obesity than normal waist circumference, higher for hypertensive than normotensive and higher in urban areas than rural ones, respectively. Our results suggest that greater focus on prevention of diabetes are necessary for obesity, central obesity, hypertensive and in urban areas among middle-aged and older in China.

## Supporting Information

S1 Table Data coding and variable description. (XLSX)
